# Re-examination of the role of endoreduplication on cell-size control in leaves

**DOI:** 10.1007/s10265-019-01125-7

**Published:** 2019-07-18

**Authors:** Hirokazu Tsukaya

**Affiliations:** 1 Department of Biological Sciences, Graduate School of Science, The University of Tokyo, Tokyo, 113-0033 Japan; 2 ExCELLS, National Institutes of Natural Sciences, Okazaki, 444-8787 Japan

**Keywords:** *Arabidopsis thaliana*, Cell size, Endocycle, Endoreduplication, Leaf, Re-examination

## Abstract

Many *Arabidopsis thaliana* genes have been reported to affect plant cell size by regulating the level of endoreduplication, which is a modified cell cycle. However, the role of endoreduplication on the altered cell size in these reports must be reconsidered based on a number of findings. First, not all plant species exhibit endoreduplication, which indicates that endoreduplication-driven cell size regulation is not universal among plants. Second, while ploidy level and cell size are correlated in the epidermal pavement cells of Arabidopsis leaves, the size of mesophyll cells appears to be comparatively uniform regardless of whether there is heterogeneity in the ploidy level. Third, changes in the cell sizes reported in mutant and transgenic Arabidopsis seem to be too large to be solely the result of altered endoreduplication level. Fourth, compensated cell enlargement, which is triggered by a severe decrease in cell proliferation in Arabidopsis leaves, is usually independent of altered endoreduplication. We re-examined the role of endoreduplication on cell-size regulation in Arabidopsis, mainly in leaves, and revealed biases in the previous studies. This paper provides an overview of the work carried out in the past decade, and presents rationale to correct the previous assumptions. Based on the considerations provided in this report, a re-examination of previous reports regarding the roles of mutations and/or transgenes in the regulation of cell size is recommended.

## Introduction

Cell-size regulation is a fundamental control system for organogenesis. Leaves are a determinate organ in plants, and leaf size is dependent on both the size and number of cells in the leaf (Tsukaya 2008). Melaragno et al. (1993) reported that cell size and the level of endoreduplication, a specialized cell cycle that causes duplication of the nuclear genome in each cycle, were correlated in the pavement cells of the leaves of *Arabidopsis thaliana* (Arabidopsis, hereafter). Subsequently, many studies focused on the role of endoreduplication on cell-size control in Arabidopsis (reviewed in Breuer et al. 2010; Sugimoto-Shirasu and Roberts 2003). In many cases, an endoreduplication-dependent ploidy increase has been found to contribute to enhanced cell expansion, as demonstrated in etiolated hypocotyls (Jakoby and Schnittger 2004), giant cell differentiation in the sepal epidermis (Roeder et al. 2010), and the cell elongation process in the elongation zone of roots (Bhosale et al. 2018; Petricka et al. 2012). While I agree that endoreduplication has a role in cell-size regulation in Arabidopsis, I believe that this role has been overestimated. The role of endoreduplication in enhanced cell expansion should be reconsidered based on a number of findings. Of these, the most important are as follows:

1. Although the role of endoreduplication has been extensively studied in Arabidopsis, many plant species, such as rice, lettuce, and peppermint, do not exhibit endoreduplication in their organs (Barow and Meister 2003; Fig. 1). Even in these other plant species, cell-size variation is observed. Thus, endoreduplication is not the general mechanism by which variations in cell size occur. This is also the case in animals, as Ullah et al. (2009) wrote: “in contrast with arthropods, developmentally regulated endoreduplication in mammals is rare. The only well characterized example is differentiation of trophoblast stem (TS) cells into trophoblast giant (TG) cells.” Indeed, our (human) body does not exhibit endoreduplication. In other words, although endoreduplication system is widely seen in multicellular organisms, endoreduplication-dependent developmental processes are not universal.

**Fig. 1:**
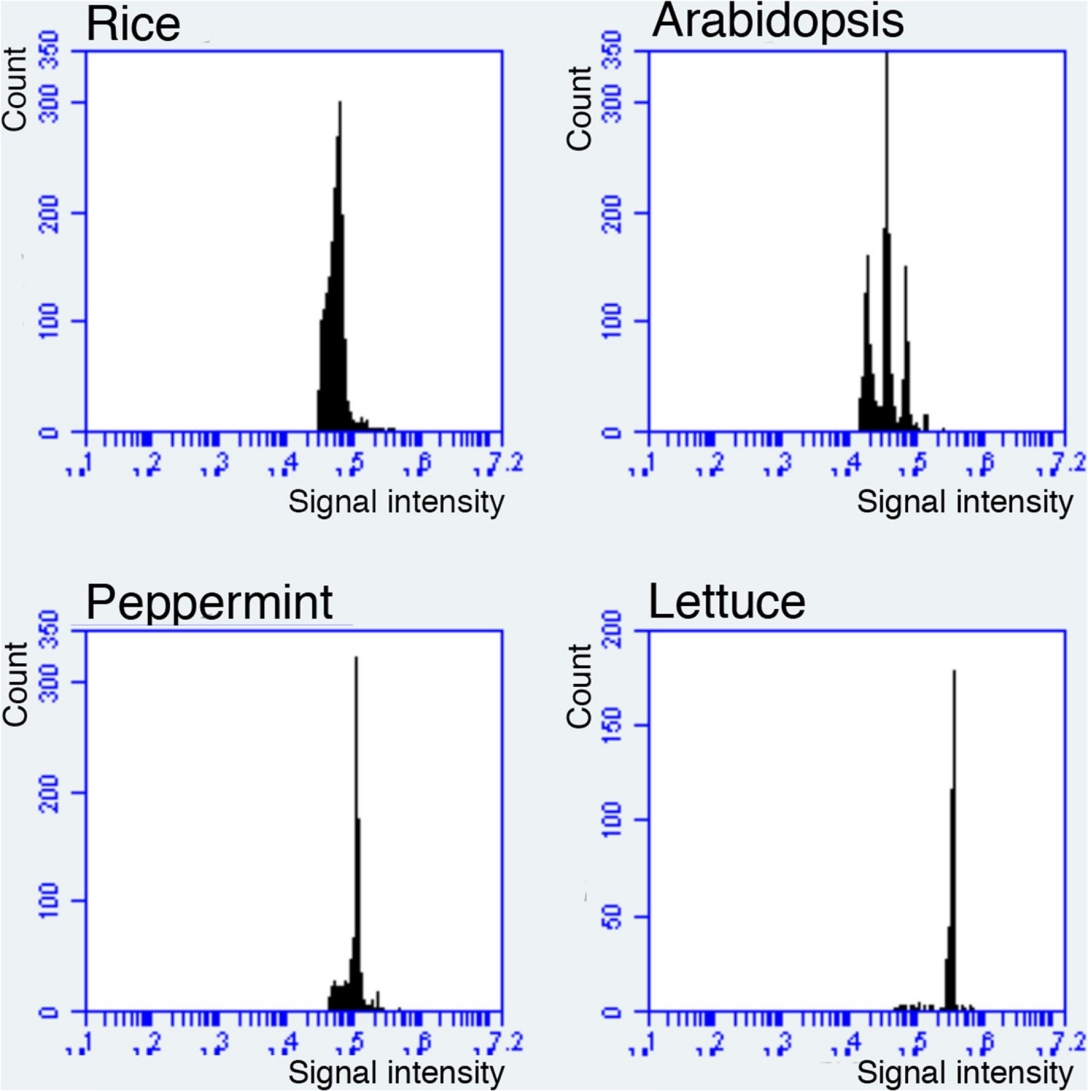
Nuclear ploidy distribution in leaves of some angiosperm species. Mature leaves of rice, Arabidopsis, peppermint, and lettuce were analyzed as described in Kozuka et al. (2005) using a flow cytometer (BD FACS AriaII or Accuri C6; Becton–Dickinson, USA). The *x*-axis indicates the signal intensity of propidium iodide, which reflects the nuclear DNA content. Note that only Arabidopsis exhibits endoreduplication and the other three species do not
2. Melaragno et al. (1993) found that a mosaic cell size distribution in the pavement cells of leaves was due to a mosaic occurrence of repeated cycles of endoreduplication. For instance, some cell populations remained at the 2C (diploid) state, while some progressed to 4C (autotetraploidy) after one cycle of endoreduplication, and some proceeded further to ploidy levels of 8C, 16C, and 32C via repeated endoreduplication, as shown in Fig. 1. As a result, a mixture of very small, small, medium, large, and very large cells is formed in the epidermal layer (Fig. 2a). It is time-consuming to analyze the level of endoreduplication in situ for each cell; thus, many studies analyzed the level of endoreduplication using flow cytometry data from whole-leaf nuclei, which are extracted by chopping the leaf blade. Because endoreduplication is not synchronized, the effect of a specific gene on the level of endoreduplication is observed as a shift of the ploidy levels of some populations, as shown in Fig. 3. Quantitative evaluation using this method is difficult, as discussed below regarding the validity of the endoreduplication index (EI). Furthermore, these studies often assume that the whole leaf tissue comprises a mosaic mixture of cells with different endoreduplication levels, rather than only the epidermis. However, unlike pavement cells, palisade cells are uniform in size (Fig. 2b).

**Fig. 2:**
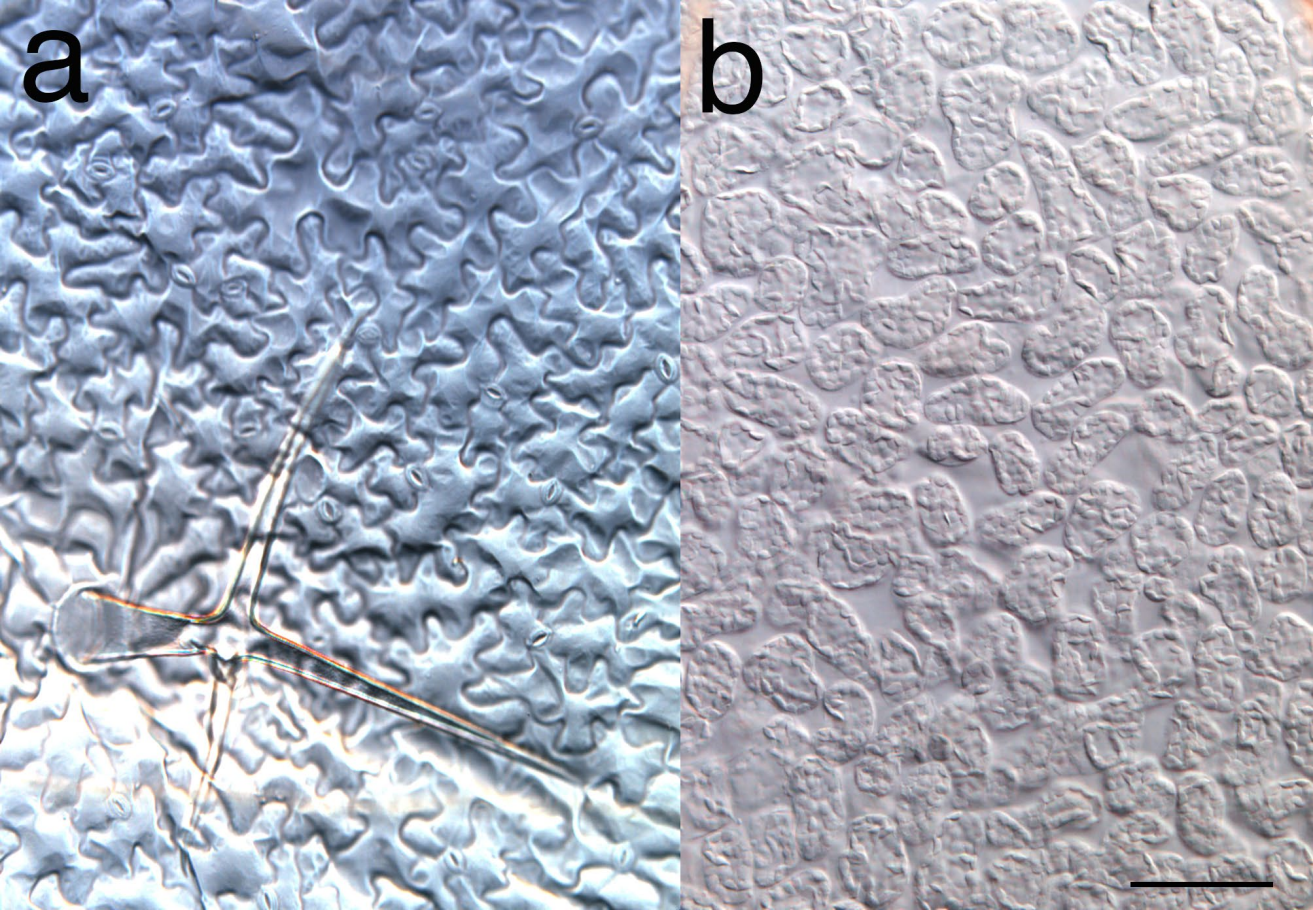
Comparison of cell-size variations in the epidermis and palisade layers of Arabidopsis leaves. Foliage leaves of Arabidopsis were fixed with FAA (5% (v/v) acetic acid, 45% ethanol, and 5% formaldehyde) and made transparent using a chloride hydrate-based solution as described earlier (Tsuge et al. 1996). Photographs were taken under a microscope equipped with a differential interference contrast system (DM4500; Leica, Germany). Note a large variation in the cell size in the epidermis (**a**), from the smallest stomata (2C) to trichomes (32C), whereas the cell size is quite uniform in the subepidermal, palisade layer (**b**). Bar, 100 μm

**Fig. 3:**
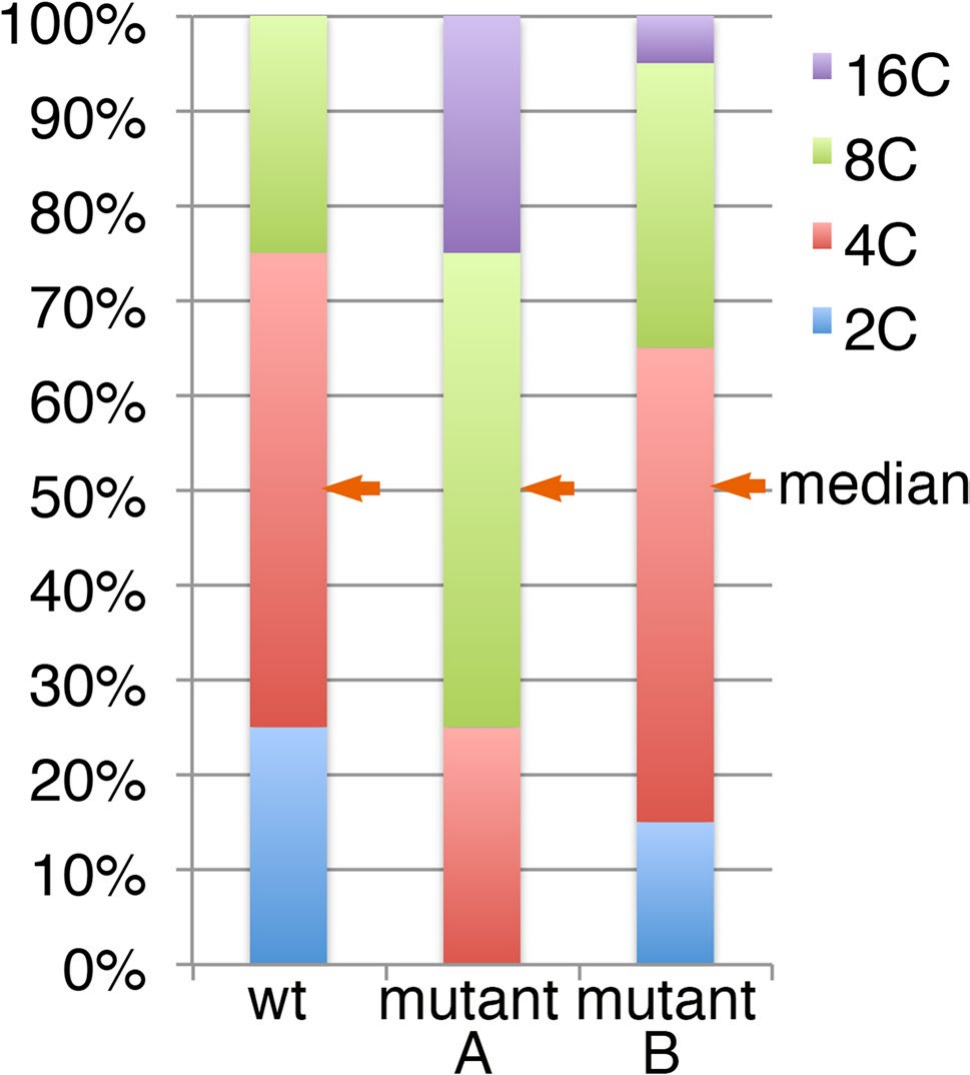
Virtual examples of the endoreduplication profile of the wild type (left) and mutant (right). Proportions of 2C, 4C, 8C, and 16C nuclei from a sample are compared with the wild type (wt). In the ‘mutant A’ line, one additional endocycle is assumed to have occurred in every cell compared to the wt. Even in this extreme case, the change in the average cell size from the wt, in terms of the projected cell area, is calculated to be only a 1.59 (= 2^2/3^)-fold increase. In most reported cases, however, the change in the endoreduplication level is much more subtle, as shown in the hypothetical ‘mutant B’. In mutant B, the median value of the nuclear ploidy level is the same as that in the wt
3. In the compensation phenotype, which is defined as having an abnormally enhanced level of cell enlargement associated with a severe decrease in the cell number in leaves (Hisanaga et al. 2015; Tsukaya 2002), we found that the enhanced cell expansion is usually not associated with enhanced endoreduplication in mutant or transgenic Arabidopsis (Ferjani et al. 2007). Thus, even in Arabidopsis, cell enlargement can be accelerated without alterations in the levels of endoreduplication. For instance, a *Kip*-*RELATED PROTEIN 2* (*KRP2*) overexpression line exhibits very large cells in the leaf mesophyll tissue, while the endoreduplication level is decreased (De Veylder et al. 2001, 2002; Sizani et al. 2019).
4. Additionally, the cell size changes in many of these reports are too large to be the result of altered endoreduplication level. The volume of the cells generally doubles after a duplication in ploidy level (Fankhauser 1952; Gates and Goodwin 1930; Kostoff 1938; Müntzing 1936; Storchová et al. 2006). Thus, if all cells experienced one additional round of endoreduplication, as shown in the middle of Fig. 3 (‘mutant A’), the mean cell size would be expected to be doubled in cell volume. Even in this extreme hypothetical case, the increase in the projected cell area of the paradermal plane would be expected to be only 1.58 (= 2^2/3^)-fold. However, in nearly all reports, leaf cells exhibit a much subtler change in endoreduplication level. For example, in ‘mutant B’ in Fig. 3, the expected cell area increase is expected to be only 1.14-fold, as discussed below, while the change in cell size in the projected cell area in this type of mutation is often two times or greater. A more detailed calculation is discussed below.

Furthermore, the seemingly well-known relationship between endoreduplication level and cell size is not fully understood (Tsukaya 2008). It is unclear whether cell size is always proportional to ploidy level. Considering the above, I have planned three distinct studies to re-examine the role of ploidy level in cell-size regulation. This report provides an overview of these results, revealing incorrect assumptions that have been made in this area of research.

## The impact of ploidy level is different between genotypes and cell types

**Fig. 4:**
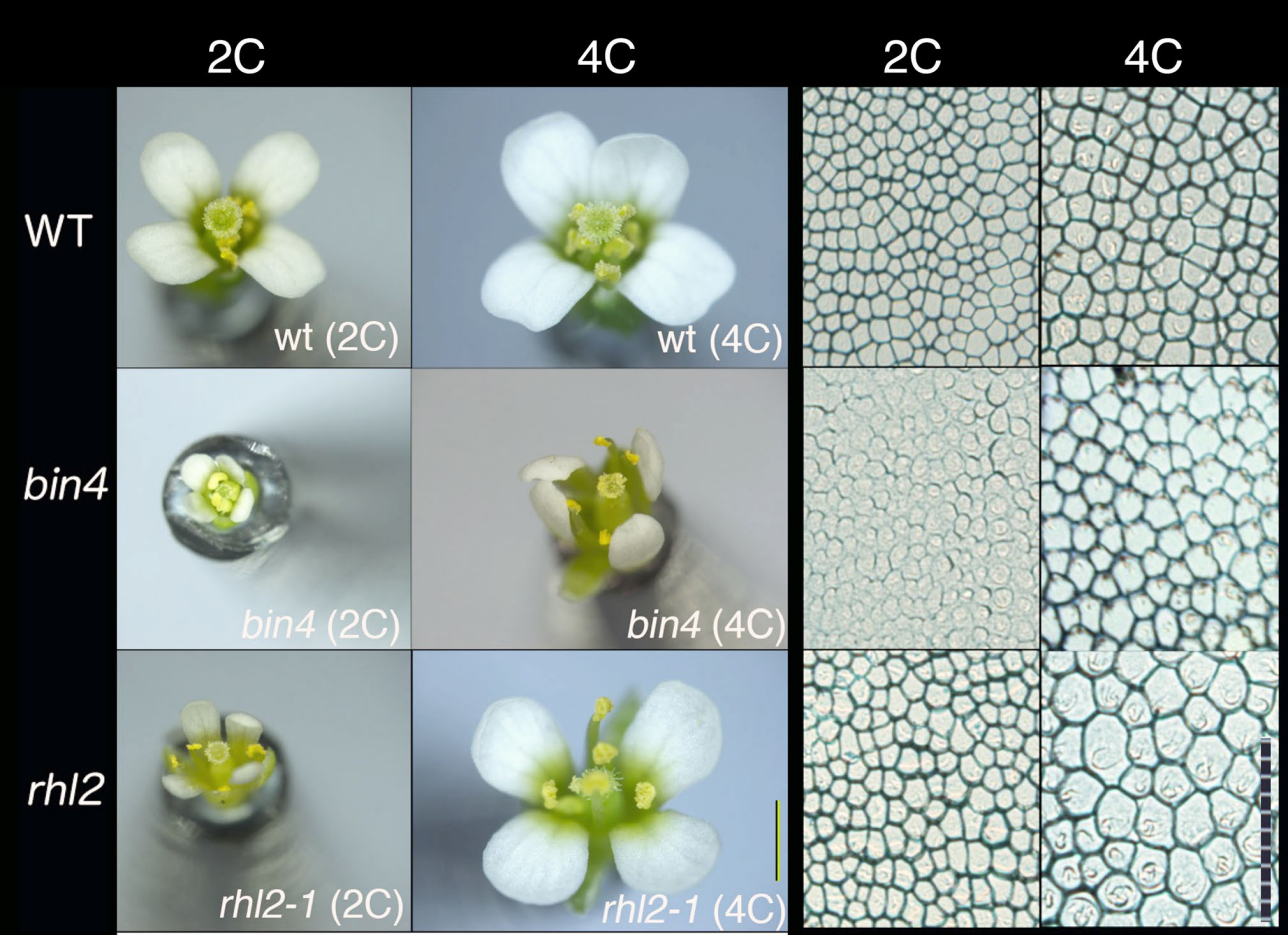
Comparative images of the diploid and tetraploid wild type, and the *bin4* and *rhl2* mutants of Arabidopsis. On the left, a whole flower is shown for each strain (bar, 1 mm) and on the right, a microscopic image of the petal epidermis is shown for each (scale, 100 μm). Note a significant increase in the petal size and cell size after autotetraploidization (from 2C to 4C) in the *bin4* and *rhl2* mutants compared with the wild type (wt)

First, I examined whether the effect of autotetraploidization on cell size is constant. The induction of autotetraploidization in plants by treatment with colchicine results in the doubling of cell volume in many species (e.g., Blakeslee and Avery 1937; Levan 1939). However, my analyses of endoreduplication-defective mutants of Arabidopsis, *brassinosteroid insensitive 4* (*bin4*) and *root harless 2*-*1* (*rhl2*-*1*, also called *atspo11*), revealed that the effect of autotetraploidization on these mutants was far larger than that on wild type (Breuer et al. 2007). Both *bin4* and *rhl2*-*1* are very small in stature, which is associated with a severe defect in the endoreduplication process (the lines have only 2C, 4C, and 8C cells in the leaves, while the wild type has cells with ploidy levels from 2C to 32C). We investigated whether an autotetraploidization, which results in the doubling of the basal ploidy level (from diploid to tetraploid), could recover plant organ growth. After the autotetraploidization, the *bin4* and *rhl2* cells were able to reach up to 16C-equivalent ploidy in the leaves. Surprisingly, the autotetraploidized *bin4* and *rhl2* plants demonstrated great recovery in stature (Breuer et al. 2007; Tsukaya 2013). Because autotetraploidization resulted in the doubling of cell volume, the increase in the leaf area base was only 1.58 (= 2^2/3^)-fold in the wild type. However, in *bin4* and *rhl2*, the increase in leaf area was more than two-fold (Tsukaya 2013). Similarly, petal size showed greater recovery in *bin4* and *rhl2* than in wt (Fig. 4).

There are at least two hypotheses that may explain this result: (1) a certain high-level ploidy state, such as 16C, is required for normal organ growth in Arabidopsis; and (2) autotetraploidization has a stronger effect on cell size in a particular genotype than in wild-type plants. The first interpretation assumes some qualitative change in the nature of cells with a high ploidy state, such as synthesis of growth factor(s) that are required for normal organ growth. If this were correct, haploid plants would demonstrate the severe defects in growth that are present in the *bin4* and *rhl2*-*1* mutants; however, this is not the case (Ravi et al. 2014). Thus, autotetraploidization must have a stronger effect on cell size in these mutants.

In wild-type Arabidopsis, autotetraploidization resulted in a 1.7-fold increase in the projected cell area on the paradermal plane in the subepidermal palisade cell layer. This value is near the expected 1.58-fold increase in cell volume, based on the assumption that cell volume is doubled by tetraploidization (Fig. 4). Conversely, the increase in this area in the *bin4* and *rhl2*-*1* mutants was 2.6- and 2.7-fold, respectively, indicating that autotetraploidization has a stronger effect on cell size in these genotypes. More studies are necessary to determine why the effect of autotetraploidization is so different between the wild type and the *bin4* and *rhl2* lines. To generally assess the effect of autotetraploidization on cell size, I performed a series of autoploidization experiments on mutants and transgenics to compare the effects of autotetraploidization in these lines (Tsukaya 2013). Surprisingly, there was a large variation in the effect of autotetraplodization on the projected cell area in the paradermal plane of the subepidermal palisade cell layer of leaves, which ranged from a 1.1- to 2.9-fold increase (Tsukaya 2013). Moreover, the difference in the effect of autotetraplodization on the projected cell area between genotypes was also seen in the petal epidermis and pollen grains. Interestingly, the effect of autotetraplodization on the projected cell area differed between these cell types, even in a given genotype (Tsukaya 2013). This finding strongly indicates that cell size is not passively proportional to ploidy level; rather, the effect of autotetraplodization on the projected cell area is regulated by genetic system(s).

## The cell size of mesophyll cells is not affected by ploidy level as much as that of epidermal cells

The effect of autopolyploidization via endoreduplication on cell size has been extensively studied in Arabidopsis. Analysis by flow cytometry has become a standard method to examine mutants or transgenics with altered cell sizes in leaves. Typically, these studies provide the average size of the leaf cells and a profile of endoreduplication events obtained from whole-leaf analysis by flow cytometry. While the original study demonstrating the correlation between cell size and endoreduplication investigated epidermal cells (Melaragno et al. 1993), most studies have measured cell size of the subepidermal palisade cells, which have a simple round shape, whereas pavement cells have complex cell shapes like pieces of a jigsaw puzzle. However, although pavement cells comprise a chimeric mixture with different cell sizes, palisade cells appear to be uniform in cell size (Fig. 2). Because flow cytometry analysis is conducted with samples obtained by chopping up the whole leaf blade, the majority of the nuclei analyzed by this method come from mesophyll cells. Thus, the observed endoreduplication profiles are primarily obtained from mesophyll cells. It must be determined whether an endoreduplication-dependent ploidy increase has an impact on cell size also in mesophyll cells. To investigate this, we analyzed Arabidopsis leaves using flow cytometry after distinguishing epidermal cells from mesophyll cells (Katagiri et al. 2016). The data revealed that the endoreduplication profiles are very similar between epidermal and mesophyll tissues. This result indicates that the effect of the endoreduplication-driven increase in ploidy level differs between epidermis and palisade cells.

**Fig. 5:**
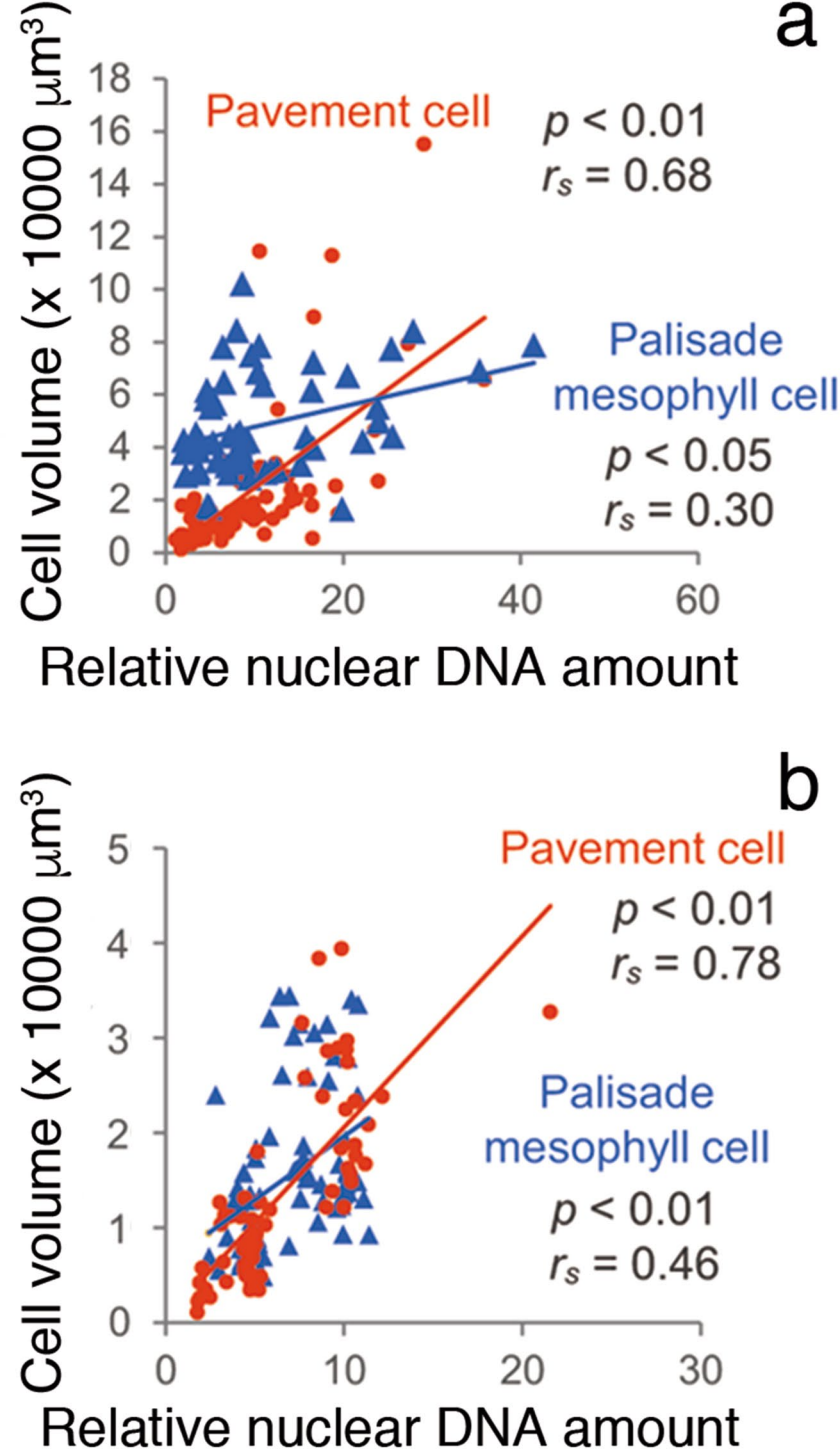
The relationship between nuclear ploidy level and cell size in the epidermis and palisade layer of Arabidopsis, before and after induction of ATML1, a master regulator of the epidermis identity in all leaf cells. The relationship between the relative nuclear DNA content (ploidy level: *x*-axis) and cell volume (*y*-axis) is shown for individual pavement and palisade mesophyll cells in a 21-day-old proRPS5A-ATML1 plant, after 14 days of control dimethyl sulfoxide treatment (**a**) and inductive ß-estradiol treatment (**b**). *r*_*s*_ indicates the Spearman rank correlation coefficient. Figure modified from Katagiri et al. (2016)

We then performed an in situ single-cell-level analysis of the ploidy level and cell size of epidermal and subepidermal palisade cells of Arabidopsis leaves (Katagiri et al. 2016). As expected, the single-cell-level measurements revealed that there was no clear correlation between cell size and ploidy level in the subepidermal layer, contrary to the findings in epidermal pavement cells (Fig. 5a). Interestingly, the correlation became much clearer even in the subepidermal layer upon ectopic expression of ATML1, a transcriptional regulator that gives cells identity of epidermis (Katagiri et al. 2016). This suggests that the correlation between ploidy level and cell size is actively regulated by a genetic system or systems that are linked to epidermal identity (Fig. 5b), or that the relationship is suppressed in the subepidermal tissue by some mechanism. In sum, both of the abovementioned studies indicate that ploidy level is related to cell size in a tissue-specific and genetic-background-dependent manner.

In addition, we determined how the initiation of the additional endocycle is regulated in mesophyll cells. Previously, it was shown in sepals that the rate of entry into the endocycle is different each time (Roeder et al. 2010). We examined the frequency of each ploidy level in leaf cells and found that the dynamics could be explained by the Poisson distribution, which differs from the abovementioned findings in sepals (Kawade and Tsukaya 2017). Interestingly, the regulatory system for repeated endoreduplication differs between foliage leaves and sepals.

## Assessment of whether the EI adequately expresses the level of endoreduplication

Considering the above, it is essential to re-examine previous reports regarding the relationship between altered endoreduplication profiles and altered cell sizes. First, all of the studies investigating the relationship between altered endoreduplication and altered cell size in leaves, whether by mutations or transgenes, revealed parallelism between the two, although only in a qualitative manner. The quantitative effects of endoreduplication on cell size are not generally discussed. However, we need such data to determine whether the relationship is correlational or causative. Second, due to the mosaic occurrence of endoreduplication cycles, all of the past studies calculated the mean cell size and EI, which is the mean of the number of cycles of endoreduplication, to evaluate correlation of the changes in cell size and the endoreduplication level. However, the mean value does not fully represent these phenomena, as it does not adequately quantify the level of endoreduplication, as discussed below.

**Fig. 6:**
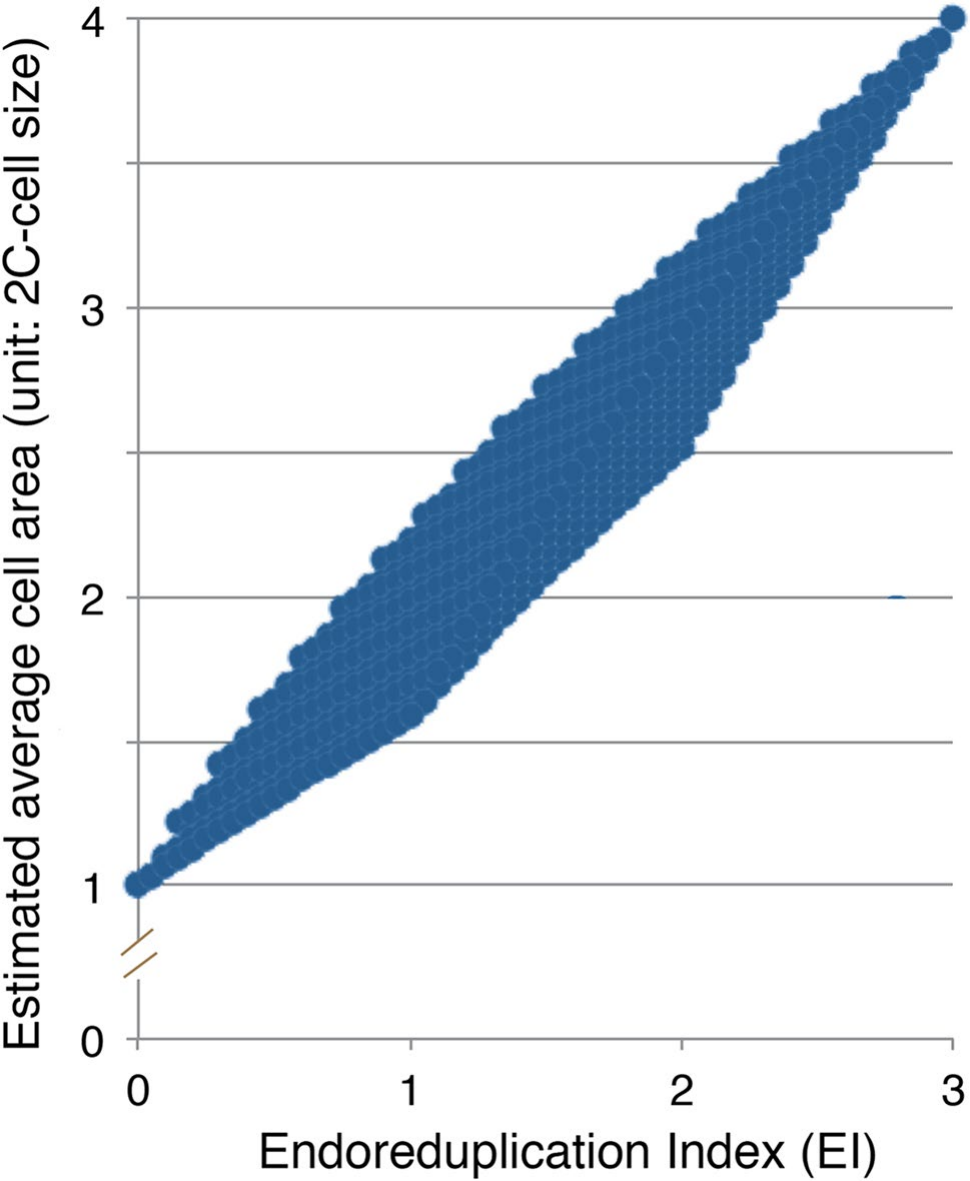
Possible combinations of expected cell size and endoreduplication index. All possible combinations of the varied ratios between 2C, 4C, 8C, and 16C cells were examined at a resolution of 5%, and then the endoreduplication index (EI) and expected cell area were calculated as described in the text. Note that EI does not accurately reflect the impact of the endoreduplication profile on the average cell size. For instance, a given strain with EI = 1.5 may demonstrate a wide range of average cell size (about 2- to 2.7-unit cell size)

Many studies have used the assumption that the EI value is proportional to the average cell size, but this is not always correct. For example, the same EI would be calculated in the following two scenarios: (a) 50% 2C cells and 50% 8C cells; and (b) 100% 4C cells; however, the expected average cell size would be different. In these scenarios, if the cell volume of the 4C cells were two times larger than that of the 2C cells, and the cell volume of the 8C cells were two times larger than that of the 4C cells, the average cell size would have a 2.5- and two-fold of 2C cell size in scenarios (a) and (b), respectively. In real samples, because the ratios of 2C, 4C, 8C, and 16C cells are variable, there can be wide variations in the endoreduplication profile. Figure 6 shows the relationship between EI and expected cell size, and indicates that EI, which is a mean value, does not adequately express the relationship between the altered endoreduplication profile and altered cell size in a strictly quantitative manner.

To examine the relationship between the endoreduplication profile and cell size, the median value is more useful (see Fig. 3). For instance, examining the median values of ploidy level and cell size in the mesophyll cells of *fugu* series mutants and transgenics in the data reported by Ferjani et al. (2007) reveals an inconsistency between ploidy level and cell size. While all of the examined strains had the same median ploidy level in the 4C state, the median cell size significantly differed between the strains, indicating that the altered endoreduplication profile was not responsible for the altered cell size. Thus, if the median cell size of the mutants or transgenics differed from that of the wild type while the median ploide level stayed at 4C as the wild type, the altered endoreduplication profile would not be the major factor of the cell-size change.

## Before the start of endoreduplication, cell size differs between genotypes

**Fig. 7:**
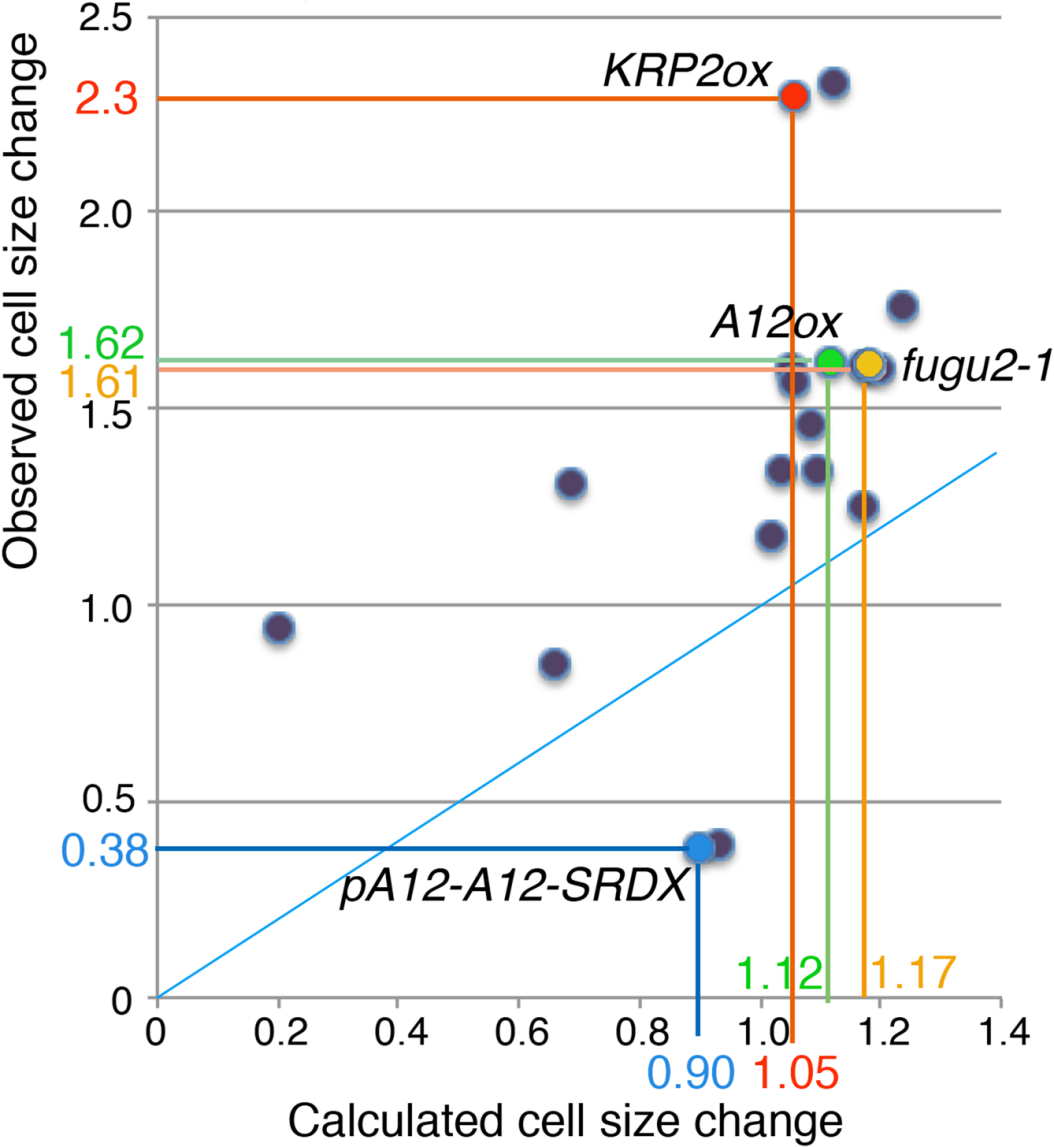
Metadata analysis of the expected and observed cell-size changes in leaf cells of Arabidopsis mutants and/or transgenics. The relationship between observed (*y*-axis) and expected (*x*-axis) changes in cell size calculated from endoreduplication profiles for palisade cells is shown. Each point represents data from a particular mutant or transgenic strain. All data are relative values against those of the wild type (wt). If the observed changes in cell size were caused solely by changes in endoreduplication, with the assumption that each endoreduplication cycle results in a doubling of cell volume, then the points would fall on the blue line. Modified from Tsukaya (2019)

To assess the degree to which altered endoreduplication contributes to observed cell-size changes, I conducted metadata analyses using data from past reports. I calculated the “expected cell-size change” based on the assumption that leaf-cell volume doubles for each endoreduplication, as discussed above (Robinson et al. 2018). The relative mean cell volume was calculated from the observed differences in the endoreduplication profiles (the ratios of 2C, 4C, 8C, 16C, and 32C), using the 2C cell volume as a unit size. Because all of the previous reports measured the projected cell area on the paradermal plane, the expected cell volume was converted into the expected cell area by calculating the [cell volume]^2/3^. As shown in Fig. 3, the expected cell-size increase in terms of cell area was calculated as 1.59-fold in ‘mutant A’ and 1.14-fold in ‘mutant B’. To account for the influences of laboratory and culture conditions, I then normalized the data by converting the cell-size data of mutants or transgenics to relative values compared to the wild type for each data set. The mean cell sizes measured in these reports were also normalized and expressed as relative values compared to the wild type for each data set. Past data were obtained from 11 publications that reported both the endoreduplication profiles and cell sizes for the leaves of the same age and position. Figure 7 shows the relationship between the expected cell-size changes determined from the endoreduplication profiles and the observed cell-size changes. It is evident from this figure that the observed and expected size changes were not perfectly correlated. In most cases, the observed cell-size change was greater than the expected change. We must consider the reasons for this discrepancy.

A key point is that these studies assume that only post-mitotic expansion, which is associated with endoreduplication, can affect the final cell size. However, this is incorrect. Let us examine the *KRP2* over-expressor (KRP2 ox) line, where endoreduplication is suppressed but cell expansion is strongly enhanced. According to Ferjani et al. (2007), the cell area of KRP2 ox was already 2.17-fold larger than that of the wild type in the mitotic phase. Based on the calculations discussed in this report, KRP2 ox was expected to have 1.05-fold larger cells due to the effects of altered endoreduplication (i.e., the overall endoreduplication level was low, but the ratio between 2C, 4C, 8C, and 16C was altered such that the mean effect of the endoreduplication on cell size was expected to be slightly positive). Interestingly, the sum of these effects (2.17 × 1.05 = 2.28) resulted in a perfect match with the observed cell-size change, which was 2.3-fold larger in area (Fig. 7). This indicates that, in addition to post-mitotic cell expansion, cell-size differences in the mitotic phase are also very important to the determination of the final cell size.

I examined other publications that reported cell size at the mitotic phase and found that the observed cell-size changes in the pA12-A12-SRDX lines (decrease) and the A12ox line (increase) (Hur et al. 2015) could also be explained by the combination of altered cell size at the mitotic phase and the expected effect of the altered endoreduplication profile (Tsukaya 2019). If we omit the effects of the changes in the diploid cell size at the mitotic phase, we cannot explain the differences between the final cell sizes of these pA12-A12-SRDX and A12ox lines and the wild type by the effect of endoreduplication (Fig. 7). However, if we combine the expected effects of the change in endoreduplication and altered cell size at the mitotic diploid phase, these differences can be explained (Tsukaya 2019). Additionally, I measured the cell size of *fasciata1/fugu2*-*1* (*fugu2*) mutant leaves, in which the discrepancy between the expected cell size change and the observed cell-size change was evident, and found that the *fugu2* mutant had a much larger cell size before the start of post-mitotic expansion (Tsukaya 2019). All of the abovementioned four cases demonstrate the importance of measuring the default cell size at the 2C mitotic phase to facilitate the discussion of the roles of genes on the cell size at maturity. In the above cases, we presume that the responsible genes affect cell size at the diploid stage before the start of endoreduplication. This viewpoint has been overlooked by the overestimation of the role of endoreduplication on cell-size regulation.

All of the previous studies demonstrated qualitative parallels between the changes in cell size and the changes in endoreduplication level. Subsequently, it was assumed that alteration of endoreduplication level was the mechanism by which many genes affected mature cell size. However, it was unclear how much of the influence of these genes on cell size occurs via changes in endoreduplication. Previously, no data had been presented to indicate whether the levels of changes in endoreduplication were related to the changes in cell size at maturity in a strictly quantitative manner. After this quantitative examination (Fig. 7), we found that the observed cell-size change was not fully explained by the observed change in endoreduplication; in addition, we should consider the default, diploid-state cell size at mitosis. Consequently, many genes that have been reported to control cell size via changes in endoreduplication likely control cell size at the default diploid stage. Given these findings, the roles of these genes must be re-examined.

## Conclusion

A number of studies have revealed that some genes affect both the endoreduplication profile and cell size in leaves. However, as discussed in this paper, the analytical methods used in these studies may have been inadequate to draw such conclusions. Based on the above discussion, the following must be taken into account when conducting studies assessing the role of endoreduplication on cell-size control in leaves: the epidermal and mesophyll cells should be distinguished; the background genotype must be considered; the median value rather than the mean value should be used for cell size and endoreduplication level; the EI should not be relied upon; and differences in cell size can occur due to genetic influence even before the start of post-mitotic expansion. Based on these considerations, we must re-examine previous reports of the role of mutations and/or transgenes on the regulation of cell size.
